# Skeletal muscle metabolism in health and disease: Mechanisms, interventions, and clinical perspectives

**DOI:** 10.1016/j.isci.2026.115024

**Published:** 2026-02-14

**Authors:** Donghai Lin, Linglin Zhang, Caihua Huang, Wei Shao

**Affiliations:** 1 Key Laboratory for Chemical Biology of Fujian Province, College of Chemistry and Chemical Engineering, Xiamen University, Xiamen, China; 2 Research and Communication Center of Exercise and Health, Xiamen University of Technology, Xiamen, China; 3 Xiamen Humanity Hospital, Fujian Medical University, Fujian, China

**Keywords:** health sciences, medicine, medical specialty, endocrinology

## Abstract

Skeletal muscle is a vital metabolic organ that regulates systemic energy homeostasis by coordinating glucose uptake, fatty acid oxidation, and amino acid metabolism. Its remarkable capacity for dynamic adaptation, termed metabolic flexibility, underpins physical performance and protects against metabolic diseases such as obesity, type 2 diabetes, and sarcopenia. This review provides an integrative synthesis of the molecular and signaling networks that orchestrate skeletal muscle metabolism, focusing on key regulators including insulin, AMPK, mTOR, and PGC-1α. We also examine how disruptions in these pathways lead to mitochondrial dysfunction, lipid dysregulation, and muscle wasting. We explore the therapeutic landscape across pharmacological, exercise-based, and nutritional interventions, emphasizing mitochondrial-targeted strategies and myokine-mediated communication as emerging modalities for restoring metabolic resilience. Additionally, we emphasize the growing importance of multi-omics technologies and inter-tissue communication in improving mechanistic understanding and advancing precision medicine. This review integrates mechanistic, translational, and clinical perspectives to underscore the importance of a systems-level approach to skeletal muscle metabolism. This approach is essential for developing targeted, multidimensional therapies aimed at enhancing metabolic health and extending healthspan.

## Graphical abstract

**Figure:**
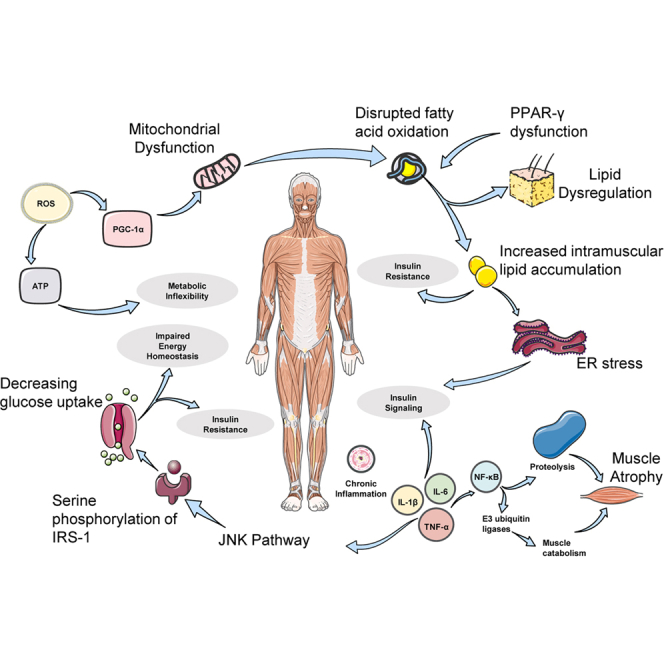

## Introduction

Skeletal muscle plays a critical role in regulating systemic metabolism, including glucose homeostasis, lipid oxidation, and amino acid metabolism. As the body’s largest organ by mass, skeletal muscle is essential for energy production and metabolic balance. In addition to enabling movement, skeletal muscle significantly impacts metabolic disorders such as obesity, type 2 diabetes (T2D), and muscle-wasting diseases.

Skeletal muscle is the primary site of postprandial glucose uptake, accounting for 70%–80% of glucose disposal via insulin-stimulated GLUT4 translocation. Disruption of this pathway contributes to the insulin resistance and hyperglycemia that are hallmark features of T2D.^1^^,^^2^ Glucagon-like peptide-1 (GLP-1), in particular, has been shown to enhance muscle glucose uptake and microvascular blood flow, highlighting its therapeutic relevance.^3^

In lipid metabolism, skeletal muscle plays a key role in fatty acid oxidation, which is essential for sustaining energy production, especially during prolonged exercise. Efficient lipid utilization improves insulin resistance. In contrast, impaired fatty acid metabolism leads to the accumulation of intramyocellular lipids, and promotes metabolic dysfunction.^4^^,^^5^ Interventions such as eicosapentaenoic acid (EPA) supplementation have been shown to increase both glucose and fatty acid uptake in myotubes derived from obese individuals.^6^

Amino acid metabolism is also critical for supporting protein synthesis and energy production in response to exercise, fasting, and illness. Skeletal muscle serves as a reservoir of amino acids for gluconeogenesis and protein turnover, with branched-chain amino acids (BCAAs) activating mTOR signaling to promote muscle growth and repair. Muscle-bone interactions further enhance glucose metabolism, emphasizing the systemic impact of muscle metabolism.^7^

Disturbances in skeletal muscle metabolism play a central role in chronic diseases. Impaired fatty acid oxidation contributes to ectopic fat deposition and exacerbates insulin resistance. Recent studies have highlighted pantothenate kinase 4 (PanK4) as a key modulator of glucose and lipid metabolism and a promising therapeutic target.^8^ Similarly, exercise improves insulin resistance by increasing glucose uptake and fatty acid oxidation, reinforcing its role as a metabolic intervention.^1^ In addition, bioactive compounds such as β-caryophyllene have been shown to benefit glucose metabolism and reduce oxidative stress in skeletal muscle.^9^

Muscle wasting conditions such as cachexia, sarcopenia, and disuse atrophy are driven by metabolic dysfunction, impaired glucose uptake, increased proteolysis, and mitochondrial dysfunction. These factors lead to progressive muscle loss. Ethyl 3-hydroxybutyrate (3-HB), a ketone ester, has emerged as a promising intervention for cancer cachexia, as it increases serum 3-HB levels, reduces tumor-induced muscle atrophy, and improves survival.^10^ 3-HB mechanistically enhances the TCA cycle activity, stimulates protein synthesis, and inhibits proteolysis, thereby promoting metabolic stability and reducing oxidative stress. These findings suggest that ketone supplementation could help preserve muscle mass in cancer patients, eliminating the need for strict dietary interventions. In addition, lactate has demonstrated therapeutic potential in mitigating muscle wasting, emphasizing the importance of targeting metabolic pathways to combat muscle wasting disorders.^11^ Fucoxanthin supplementation has also shown efficacy in preserving muscle mass and improving insulin resistance, reinforcing the therapeutic relevance of skeletal muscle metabolism.^12^

Beyond its intrinsic metabolic role, skeletal muscle engages in dynamic communication with other organs, which profoundly influences systemic energy homeostasis. Recent metabolomics-driven studies of cancer cachexia have identified key metabolic changes and revealed new therapeutic targets, especially those related to remodeling of lipid metabolism and restoration of insulin sensitivity.^13^ Advances in metabolomic profiling have deepened our understanding of the metabolic disorders underlying cachexia. These advances have revealed disrupted energy pathways and maladaptive substrate utilization that drive progressive muscle wasting. These studies have identified distinct metabolites and dysregulated metabolic circuits as promising biomarkers for early cachexia detection and precise management.^14^

Moving forward, the combination of integrative metabolomics and precision medicine shows great potential for translating these insights into clinical applications. Future research integrating comprehensive metabolic mapping with personalized genetic and phenotypic profiling holds the potential to advance the development of precision interventions aimed at restoring metabolic homeostasis, preserving skeletal muscle integrity, and preventing cachexia-related muscle wasting.

In addition to its fundamental role in movement, skeletal muscle plays a central role in regulating systemic metabolic health by integrating energy production, substrate utilization, and inter-organ communication. This review is guided by the central premise that the ability of skeletal muscle to switch between using carbohydrates, lipids, and amino acids (i.e., metabolic flexibility) is regulated by an integrated signaling network that includes AMPK, mTOR, and PGC-1α. These interconnected pathways orchestrate cellular adaptations in response to nutrient availability, exercise, and metabolic stress. This preserves mitochondrial efficiency, energy homeostasis, and overall metabolic resilience. Moreover, the review critically examines the molecular evidence underlying this regulatory network, clarifying how its disruption contributes to major pathophysiological states such as insulin resistance, lipid dysregulation, and muscle atrophy.

Furthermore, this review integrates mechanistic evidence from both preclinical and human studies to identify key points of convergence and divergence that shape translational interpretation. By adopting this analytical and comparative framework, the review moves beyond descriptive synthesis to offer a comprehensive, systems-level perspective on skeletal muscle metabolism in both health and disease. This integrative approach aims to inform the development of targeted therapeutic strategies that enhance mitochondrial function, restore metabolic flexibility, and ameliorate chronic metabolic disorders through coordinated modulation of the AMPK-mTOR-PGC-1α signaling network.

### Literature search and selection criteria

This narrative review synthesizes evidence from a broad body of literature to construct a cohesive, mechanistically grounded framework of skeletal muscle metabolism. To ensure comprehensive and representative coverage, we conducted systematic searches of major scientific databases (PubMed, Scopus, and Web of Science) using combinations of keywords, including “skeletal muscle metabolism,” “insulin resistance,” “AMPK,” “mitochondrial biogenesis,” “PGC-1α,” “myokines,” “exercise metabolism,” “sarcopenia,” and “cachexia.” The search was restricted to peer-reviewed articles published in English up to December, 2025.

We included original research articles, meta-analyses, and authoritative reviews that provided mechanistic insights or clinical evidence relevant to metabolic regulation, pathophysiology, and therapeutic interventions in skeletal muscle. Studies were excluded if they did not directly focus on skeletal muscle, lacked peer review (e.g., conference abstracts), or presented significant methodological limitations affecting data reliability.

The final selection of references was guided by their relevance to the central hypotheses of this review, with priority given to seminal contributions and recent, high-impact studies. This approach ensured balanced representation of foundational concepts and emerging discoveries, providing a robust evidence base for the integrative analysis that follows.

## Skeletal muscle metabolism

Skeletal muscle is the largest metabolic organ in the human body, accounting for approximately 40% of total body mass. It serves as the principal site for insulin-stimulated glucose uptake, lipid oxidation, and protein turnover, thereby playing a central role in maintaining systemic energy homeostasis.

**Figure 1:**
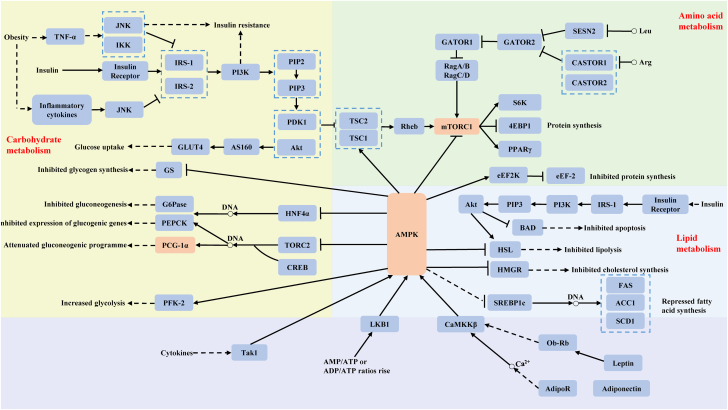
Overview of skeletal muscle substrate metabolism and core regulatory integration This schematic diagram illustrates the central metabolic networks within skeletal muscle, highlighting the interconversion and utilization of primary substrates (glucose, fatty acids, amino acids, lactate, and ketone bodies) that sustain energy homeostasis. It emphasizes the integrative role of key regulatory nodes (e.g., AMPK, mTORC1, and PGC-1α) in coordinating metabolic flexibility. The pleiotropic actions of insulin on these pathways and its modulation by hormones, myokines, and cytokines are discussed in detail in the text (see Sections glucose metabolism and hormonal and cytokine regulation of skeletal muscle metabolism) and summarized in Table 2.

This substantial mass underlies the muscle’s remarkable metabolic plasticity, enabling dynamic adaptation to physiological demands such as exercise, fasting, and nutrient fluctuations. Skeletal muscle maintains metabolic flexibility, the capacity to switch efficiently among energy substrates including glucose, fatty acids, amino acids, lactate, and ketone bodies (Figure 1). At the molecular level, these adaptive processes are coordinated by key signaling networks such as AMPK, mTORC1, and PGC-1α, which integrate energy status, substrate availability, and mitochondrial function to balance anabolic and catabolic processes.

At the molecular level, the metabolism of skeletal muscle is governed by the tightly regulated interplay of nutrient-sensing signaling pathways, mitochondrial oxidative capacity, and hormonal inputs. Key regulators include AMPK, mTORC1, and PGC-1α. These regulators coordinate mitochondrial biogenesis, oxidative phosphorylation, and the anabolic-catabolic balance. These pathways regulate essential metabolic processes, including glycolysis, fatty acid β-oxidation, amino acid catabolism, and ketone utilization. This ensures the production of sufficient ATP and maintains cellular homeostasis.

Disrupting this finely tuned metabolic network can lead to various pathological conditions, including insulin resistance, T2D, obesity, sarcopenia, and metabolic syndrome. Impaired mitochondrial function, oxidative stress, and chronic inflammation can lead to impaired substrate utilization, reduced metabolic flexibility, and exacerbated energy imbalance.

Furthermore, skeletal muscle acts as an endocrine organ by releasing myokines, such as IL-6, irisin, and FGF21, and by responding to systemic hormones and cytokines, including insulin, glucagon, cortisol, catecholamines, and proinflammatory mediators, including TNF-α and IL-1β. These factors modulate substrate transport, energy expenditure, and metabolic gene expression. This links skeletal muscle metabolism to broader systemic effects on the liver, adipose tissue, and pancreas.

This section provides a comprehensive overview of skeletal muscle metabolism, including substrate-specific pathways, mitochondrial energetics, and hormonal regulation. Understanding these processes mechanistically is essential for identifying therapeutic targets to enhance skeletal muscle function and promote systemic metabolic health.

### Skeletal muscle substrate metabolism: Glucose, lipids, amino acids, lactate, and ketones

Skeletal muscle metabolism exhibits high metabolic flexibility, shifting between different substrates based on nutrient availability, energy demand, and hormonal regulation (Figure 1). Essential metabolites and their roles in glucose metabolism are comprehensively outlined in Table 1.

**Table 1:** Key circulating substrates and intermediates in skeletal muscle glucose metabolism

Metabolite	Source	Function in skeletal muscle metabolism	Impact on glucose regulation & muscle health	Therapeutic relevance	Reference
Glucose	Dietary carbohydrates, glycogenolysis	Primary fuel source for ATP production via glycolysis. Stored as glycogen for later use.	Essential for energy homeostasis. Impaired uptake contributes to insulin resistance, type 2 diabetes, and metabolic syndrome.	Exercise, insulin therapy, SGLT2 inhibitors	DeFronzo et al.^15^; Abdulla et al.^3^
Fatty acids	Adipose tissue lipolysis, dietary lipids	β-oxidation in mitochondria generates ATP, particularly during fasting and endurance exercise.	Essential for muscle endurance and metabolic flexibility. Excess lipid accumulation is linked to insulin resistance.	Omega-3 fatty acids, caloric restriction, exercise	Jensen et al.^16^; Wu et al.^12^
Amino acids (BCAAs)	Dietary protein, muscle protein breakdown	Leucine stimulates mTORC1, supporting protein synthesis and muscle repair. Used in gluconeogenesis.	Regulates muscle growth and recovery. Excessive levels may be linked to insulin resistance.	BCAA supplementation, resistance training	Anthony et al.^17^; Cui et al.^13^
Lactate	Anerobic glycolysis in muscle fibers	Acts as a fuel source via the Cori cycle, supporting ATP production.	Enhances metabolic flexibility and mitochondrial function. Elevated levels indicate anerobic stress and fatigue.	High-intensity interval training (HIIT), lactate metabolism modulators	Brooks^18^; Zhou et al.,^19^
Ketone bodies	Hepatic ketogenesis during fasting, Low-carb diets	Alternative energy source for muscle metabolism, sparing glucose utilization.	Supports energy homeostasis during fasting. Therapeutic potential in insulin resistance and neuroprotection.	Ketogenic diet, exogenous ketone supplements	Newman et al.^20^; Zhou et al.^10^

This table summarizes key circulating substrates and intermediates central to glucose metabolism in skeletal muscle, highlighting their muscle-specific functions, impact on systemic glucose regulation, and therapeutic potential. Detailed pathways of their systemic synthesis and inter-organ metabolism are discussed in the main text (see Sections glucose metabolism, fatty acid metabolism, and lactate and ketone body metabolism).

#### Glucose metabolism

Glucose is the principal energy substrate for skeletal muscle, particularly during high-intensity exercise. Skeletal muscle accounts for approximately 70%–80% of postprandial glucose disposal, a process primarily mediated by insulin-stimulated translocation of GLUT4 transporters to the plasma membrane (Figure 1). This insulin-responsive mechanism ensures efficient glucose uptake and storage under fed conditions, thereby maintaining systemic glucose homeostasis.

Upon insulin binding, the IRS-1/PI3K/Akt signaling cascade is activated, triggering the mobilization of GLUT4-containing vesicles to the sarcolemma in both muscle and adipose tissue.^2^^,^^21^^,^^28^ Concurrently, Akt inhibits glycogen synthase kinase 3β (GSK-3β) and activates glycogen synthase, thereby promoting glycogen synthesis and intracellular glucose storage. The pathway is tightly regulated by phosphatases such as PTEN and protein phosphatase 1 (PP1) to maintain blood glucose concentrations within the physiological range of 4.4–6.1 mM, preventing hyperglycemia through dynamic feedback control.^29^

In states of insulin resistance, which are typically associated with chronic inflammation and metabolic stress, PI3K/Akt signaling is often attenuated due to serine phosphorylation of IRS-1. This modification is driven by multiple pro-inflammatory cytokines (e.g., TNF-α, IL-1β) and the activation of stress-responsive serine/threonine kinases such as JNK and IKKβ, which are upregulated in inflammatory and nutrient-overload conditions. This results in reduced GLUT4 translocation and impaired glucose uptake. Persistent hyperglycemia exacerbates oxidative stress and mitochondrial dysfunction, impairing metabolic flexibility and resulting in an elevated respiratory quotient (RQ > 0.85). If left untreated, this pathological feedback loop may progress to β-cell failure and T2D.^30^^,^^31^

In contrast, AMP-activated protein kinase (AMPK) is activated during energy-depleted states, such as exercise or fasting, in response to rising AMP/ATP ratios. AMPK enhances GLUT4 expression via the HDAC5/SIRT1 axis, stimulates PGC-1α-mediated mitochondrial biogenesis, and upregulates CPT1. This enhances fatty acid oxidation and improves substrate flexibility.^23^^,^^32^ Chronic AMPK activation facilitates efficient switching between glucose and lipid substrates and mitigates insulin resistance. However, translational studies reveal notable discrepancies between animal and human models. For instance, AMPK activation in response to exercise or energy stress is often more robust and consistent in rodents, whereas human studies show greater variability due to factors such as age, fitness level, and metabolic health status. In insulin-resistant states, AMPK response may be blunted in humans compared to animal models, highlighting the need for cautious interpretation of preclinical data.^1^^,^^23^ Bioactive compounds such as malonyl ginsenosides enhance glucose and lipid metabolism by activating the IRS-1/PI3K/Akt and AMPK pathways concurrently, thereby improving insulin resistance.^33^

Insulin-independent glucose uptake is primarily regulated by AMPK and calcium/calmodulin-dependent protein kinase (CaMK) during muscle contraction. These kinases promote GLUT4 translocation, which occurs independently of insulin. Exercise-induced increases in muscle perfusion enhance glucose delivery and uptake. This exercise-induced increase in muscle perfusion is largely mediated by nitric oxide (NO)-dependent vasodilation. Beyond its hemodynamic role, NO also acts as a signaling molecule within the muscle fiber, directly contributing to the stimulation of glucose uptake alongside AMPK and CaMK pathways.^34^ Furthermore, fasting and prior muscular activity have a synergistic effect on insulin resistance via enhanced AMPK signaling.^35^^,^^36^

Together, these regulatory pathways including insulin signaling, AMPK activation, mitochondrial dynamics, and substrate transport, form the core architecture of skeletal muscle glucose metabolism (Figure 1). Disruption of these pathways underlies a spectrum of metabolic pathologies. Table 2 summarizes key regulatory molecules and their functional implications, highlighting their relevance as potential therapeutic targets for restoring insulin responsiveness and enhancing metabolic flexibility.

**Table 2:** Core molecular components and functions of key regulatory pathways in skeletal muscle glucose metabolism

Key regulatory pathway	Key regulator	Function	Activators/inhibitors	Activation conditions	Impact on skeletal muscle health and systemic glucose regulation	Reference
Insulin signaling pathway	IR, IRS-1, PI3K, Akt, GLUT4	Insulin binds to its receptor, initiating a cascade that activates IRS-1 and PI3K, leading to the activation of Akt and promoting GLUT4 translocation to plasma membrane. Facilitates glucose uptake and glycogen synthesis in skeletal muscle.	Activators: insulin, IGF-1 Inhibitors: TNF-α, IL-1β, JNK, IKKβ, PTEN	Postprandial state, elevated blood glucose levels	Enhances glucose uptake and utilization, contributing to postprandial glucose disposal. Promotes protein synthesis via inhibition of the FOXO pathway, reducing muscle protein breakdown. Insulin resistance disrupts this pathway, leading to impaired glucose uptake and metabolic diseases such as T2DM.	Saltiel et al.^21^; Wallberg-Henriksson et al.^22^; Abdulla et al.^3^
AMPK pathway	AMPK (a, β, y subunits), LKB1, CaMKKβ	AMPK is activated by an increase in the AMP/ATP ratio, indicative of low energy status. AMPK then phosphorylates and regulates enzymes involved in glucose uptake (GLUT4), fatty acid oxidation (acetyl-CoA carboxylase), and mitochondrial biogenesis.	Activators: exercise, energy depletion (AMP/ATP↑), metformin Inhibitors: high ATP	Exercise, fasting, energy stress (low cellular energy)	Promotes fatty acid oxidation, enhances glucose uptake, and inhibits protein synthesis in favor of energy-producing pathways. AMPK activation is a key target for exercise and pharmacological interventions (e.g., metformin) to improve metabolic health.	Hardie et al.^23^; Richter et al.^1^; Ketare et al.^6^
mTORC1 pathway	mTORC1, Rheb, Akt, TSC1/2, AMPK	mTORC1 integrates signals from amino acids, energy status (via AMPK), and growth factors (such as insulin) to promote protein synthesis and drive muscle hypertrophy.	Activators: leucine, insulin, growth factors, amino acids Inhibitors: rapamycin, energy depletion (via AMPK)	Nutrient abundance (especially amino acids), postprandial state, resistance exercise	Drives muscle protein synthesis and growth. Excessive activation can lead to insulin resistance and metabolic dysfunction.	Kimball et al.^24^; Laplante et al.^25^
Mitochondrial biogenesis	PGC-1α, NRF1/2, TFAM, AMPK, SIRT1	PGC-1α enhances mitochondrial function and energy production. Coordinates the expression of genes involved in mitochondrial DNA replication, energy production, and oxidative metabolism.	Activators: exercise, caloric restriction, PGC-1α agonists (e.g., resveratrol), AMPK activation Inhibitors: sedentary lifestyle, aging, chronic inflammation	Endurance exercise, energy stress, mitochondrial stress	Improves insulin resistance and reduces metabolic disease risk. Dysregulation is linked to insulin resistance and aging-related muscle loss.	López-Lluch et al.^26^; Cantó et al.^27^

This table summarizes the principal molecular players and primary physiological functions of major signaling pathways regulating skeletal muscle glucose metabolism. Detailed discussions of pathway activators and inhibitors (e.g., insulin, exercise, nutrients, and stress responses), contextual regulation, and interactions with additional pathways (e.g., Ca^2+^/CaMK and inflammatory kinases) are provided in the main text (see Sections glucose metabolism, mitochondrial function and energy homeostasis in skeletal muscle, **hormonal and cytokine regulation of skeletal muscle metabolism**, mitochondrial dysfunction and insulin resistance, and chronic inflammation and lipid metabolism dysregulation).

#### Fatty acid metabolism

Fatty acids serve as a crucial and sustained energy source for skeletal muscle, particularly during fasting and prolonged, moderate-intensity exercise. Their catabolism occurs predominantly via mitochondrial β-oxidation, a process essential for maintaining metabolic flexibility and energy homeostasis (Figure 1; Table 2).

The uptake of long-chain fatty acids into skeletal muscle is mediated by the translocation of the fatty acid transporter FAT/CD36 to the plasma membrane.^37^ AMPK activation, induced by energy stressors such as exercise or fasting, along with insulin signaling, facilitates this translocation and enhances fatty acid uptake.^38^ Depending on cellular energy demand, these fatty acids are either oxidized for ATP production or stored as triglycerides.^39^

However, dysregulation of fatty acid transport and oxidation leads to intramyocellular lipid accumulation, which contributes to lipotoxicity and compromises insulin signaling. Overexpression of CD36 has been strongly associated with ectopic lipid deposition in non-adipose tissues, including the liver and skeletal muscle, exacerbating insulin resistance and promoting metabolic dysfunction.^40^^,^^41^

Carnitine palmitoyltransferase 1 (CPT1), located on the outer mitochondrial membrane, is the rate-limiting enzyme responsible for transporting long-chain fatty acyl-CoA into mitochondria for β-oxidation. This process is essential for sustaining energy production during periods of low glucose availability. CPT1 activity is tightly regulated by malonyl-CoA, a glycolytic intermediate that inhibits CPT1 under fed conditions, thereby linking carbohydrate and lipid metabolism.^5^^,^^42^

Exercise and pharmacological activation of AMPK such as by metformin, upregulate CPT1 expression and relieve malonyl-CoA-mediated inhibition. This promotes mitochondrial fatty acid oxidation and enhances skeletal muscle oxidative capacity, contributing to improved metabolic flexibility and endurance.^43^^,^^44^

In contrast, impaired CPT1 function or fatty acid oxidation leads to the cytosolic accumulation of lipotoxic intermediates, including diacylglycerols (DAGs) and ceramides. These metabolites disrupt insulin signaling primarily by activating serine/threonine kinases such as PKCθ and by engaging inflammatory pathways like NF-κB, which promote inhibitory serine phosphorylation of IRS-1. Furthermore, mitochondrial overload from unmetabolized fatty acids increases reactive oxygen species (ROS) generation by 30%–50%, damaging the electron transport chain (ETC) and compromising ATP synthesis. It is critical to note that the activation of these and other serine/threonine kinases (e.g., JNK, IKKβ) represents a convergent mechanism in insulin resistance, being stimulated not only by lipotoxicity but also by endoplasmic reticulum (ER) stress, inflammation, and hyperglycemia itself. Thus, the convergence of lipotoxicity, mitochondrial dysfunction, and kinase-driven disruption of insulin signaling forms a critical pathological axis underlying metabolic inflexibility and disease progression.^45^^,^^46^

#### Amino acid metabolism

Amino acids are indispensable substrates for protein synthesis. Under normal physiological conditions, their direct contribution to ATP production is minimal, and they are not stored intracellularly as discrete energy reserves like glycogen or triglycerides. However, during metabolic stress such as prolonged fasting, intense exercise, or catabolic illness, amino acids derived from skeletal muscle proteolysis become critical alternative substrates. Skeletal muscle represents the largest dynamic protein reservoir in the body. Through regulated proteolysis, it can release amino acids into circulation to support hepatic gluconeogenesis, oxidative metabolism in other tissues, or the synthesis of acute-phase proteins, thereby maintaining systemic energy and metabolic balance (Figure 1; Table 2).

BCAAs, particularly leucine, play a pivotal anabolic role through activation of the mTORC1 pathway. Leucine interacts with Sestrin2 and leucyl-tRNA synthetase, leading to phosphorylation of downstream effectors such as ribosomal protein S6 kinase (S6K). This process promotes muscle protein synthesis. This pathway is particularly relevant during resistance exercise and recovery from muscle atrophy. Notably, species-specific differences in mTORC1 signaling have been observed. Rodent studies demonstrate rapid and robust activation following exercise or leucine supplementation, whereas human trials, especially in older adults or individuals with metabolic syndromes, often show more modest responses. These discrepancies may reflect variations in study design, muscle fiber composition, or hormonal context, underscoring the limitations of directly extrapolating animal data to human physiology.^25^^,^^47^ While chronic BCAA supplementation can enhance mTORC1 activity by 30%–50%, excessive intake may disrupt metabolic homeostasis and lead to adverse effects.^25^^,^^47^

Under catabolic conditions, amino acids such as alanine and glutamine undergo deamination to generate α-ketoglutarate and other intermediates that enter the TCA cycle, supporting ATP production via oxidative phosphorylation. Concurrently, hepatic gluconeogenesis utilizes the carbon skeletons of glucogenic amino acids, accounting for approximately 20% of total glucose output during fasting.^47^^,^^48^

Paradoxically, elevated plasma BCAA levels have been associated with insulin resistance and T2D. In insulin-resistant states, incomplete mitochondrial oxidation of BCAAs leads to the accumulation of toxic intermediates such as branched-chain keto acids (BCKAs). These intermediates impair insulin receptor substrate-1 (IRS-1) signaling and reduce GLUT4-mediated glucose uptake. In addition, chronic mTORC1 hyperactivation by excess BCAAs may promote lipogenesis and exacerbate metabolic syndrome.^49^^,^^50^

Therapeutic amino acid supplementation, especially of essential amino acids, has been shown to improve muscle protein synthesis and mitigate muscle loss in catabolic conditions such as cachexia, sarcopenia, and disuse atrophy. This highlights the potential of targeted amino acid supplementation in clinical metabolic interventions.^51^^,^^52^

#### Lactate and ketone body metabolism

Lactate and ketone bodies serve as critical metabolic intermediates that support energy production, maintain redox balance, and enhance metabolic flexibility in skeletal muscle and other tissues (Figure 1; Table 2).

Lactate, long considered a waste product of anerobic glycolysis, is now recognized as an essential fuel and signaling molecule. During high-intensity exercise or hypoxia, lactate is produced by glycolytic muscle fibers and transported between tissues via monocarboxylate transporters (MCT1 and MCT4), a process known as the *lactate shuttle*. This mechanism enables lactate oxidation in mitochondria-rich tissues such as the heart, liver, and oxidative muscle fibers, thereby sparing glucose and enhancing whole-body ATP efficiency.^18^^,^^53^ Moreover, lactate supports gluconeogenesis in the liver (Cori cycle), linking anerobic muscle activity with hepatic energy production. Lactate homeostasis itself is actively maintained through coordinated inter-tissue crosstalk. Systemic lactate levels are regulated by a balance between glycolytic production and its utilization or conversion, with adipose tissue lipolysis playing a key modulating role,^54^ underscoring its role as a systemic metabolic buffer.

Ketone bodies, including β-hydroxybutyrate (β-HB) and acetoacetate, are produced in the liver from fatty acids during prolonged fasting, carbohydrate restriction, or ketogenic diets. These molecules replace glucose as a primary energy source, especially for the brain and skeletal muscle under low-insulin conditions. In skeletal muscle, ketone utilization occurs through mitochondrial oxidation, where β-HB is converted into acetoacetyl-CoA and subsequently acetyl-CoA, entering the TCA cycle for ATP production.

Ketones enhance mitochondrial efficiency by lowering ROS production and upregulating energy-sensing transcription factors, including FOXO3 and PPARα. These pathways promote oxidative metabolism and exert anti-catabolic effects, preserving lean muscle mass during catabolic stress (e.g., fasting, illness, etc).^53^^,^^55^^,^^56^ Additionally, β-HB has been shown to inhibit histone deacetylases (HDACs), supporting anti-inflammatory and antioxidative responses, further contributing to skeletal muscle health.

Together, lactate and ketone metabolism play essential roles in energy redistribution and metabolic adaptation under stress or nutrient-deprived states. Their efficient regulation enhances systemic energy homeostasis and offers therapeutic potential for metabolic disorders and muscle-wasting conditions.

### Mitochondrial function and energy homeostasis in skeletal muscle

Mitochondria are the primary energy-generating organelles in skeletal muscle, responsible for oxidative phosphorylation, ATP production, and cellular redox balance. The capacity of mitochondria to efficiently oxidize glucose, fatty acids, and amino acids determines muscle endurance, metabolic resilience, and systemic energy homeostasis.^57^

#### Mitochondrial biogenesis and exercise adaptation

Mitochondrial biogenesis, the process of generating new mitochondria, is regulated by PGC-1α, which activates downstream transcription factors, including NRF1/2 and TFAM, to enhance mitochondrial DNA replication and oxidative phosphorylation capacity. Exercise, particularly endurance exercise, is a potent stimulator of mitochondrial biogenesis. It induces PGC-1α expression, thereby increasing mitochondrial content and ATP synthesis efficiency (Table 2).

Endurance exercise activates the AMPK/PGC-1α signaling pathway, which increases NRF1/2 and TFAM, thereby driving mitochondrial DNA replication and expansion of the ETC. This transcriptional cascade increases the density of mitochondria in skeletal muscle by 30%–50%, enhancing oxidative phosphorylation capacity and endurance performance.^58^^,^^59^ Despite the conserved role of PGC-1α in mitochondrial biogenesis, comparative analyses indicate significant interspecies differences. Exercise-induced PGC-1α upregulation is typically more dramatic in animal models (e.g., mice and rats), leading to substantial mitochondrial expansion. In contrast, human studies observe more variable and often subdued responses, influenced by factors like exercise intensity, genetic polymorphisms, and age-related declines. Such discrepancies emphasize the challenge of translating mitochondrial therapies from bench to bedside.^58^^,^^60^

High-intensity interval training (HIIT) elevates lactate flux, activates MCT1 transporters, and stimulates mitochondrial enzyme adaptations (e.g., increased citrate synthase activity). This remodeling improves metabolic flexibility. HIIT increases mitochondrial respiratory capacity by 20%–35% within weeks.^61^^,^^62^ Metabolic flexibility is also tested under prolonged nutrient deprivation. A human study found that seven days of fasting enhanced whole-body fat oxidation during exercise but also induced a shift toward branched-chain amino acid catabolism in skeletal muscle to support energy needs, demonstrating a complex, multi-substrate adaptation beyond simple lipid reliance.^63^

In addition to stimulating mitochondrial biogenesis, exercise robustly activates mitochondrial quality control mechanisms, preserving a healthy, functionally competent organelle network. Mitophagy, the selective removal of damaged mitochondria via autophagy, is markedly increased following endurance and resistance exercise. This process is crucial for maintaining metabolic efficiency. BCL2-regulated autophagy is essential for muscle glucose homeostasis,^64^ underscoring the metabolic significance of this pathway. Remarkably, even a single bout of resistance exercise can trigger mitophagy, which may involve the ejection of mitochondria from human skeletal muscle,^65^ This highlights the rapid and dynamic responsiveness of this surveillance system. Furthermore, PGC-1α plays a pivotal role in coordinating exercise-induced autophagy and mitophagy,^66^ demonstrating the tightly regulated balance between mitochondrial biogenesis and degradation during adaptive remodeling.

Recent studies have highlighted the importance of the mitochondrial unfolded protein response (UPRmt) in adapting to exercise. Aerobic training induces mitonuclear imbalance and UPRmt activation in the skeletal muscle of aged mice,^67^ suggesting a protective mechanism against age-related mitochondrial decline. Additionally, physical exercise activates UPRmt signaling via the c-Jun N-terminal kinase (JNK) pathway,^68^ revealing a distinct molecular mechanism by which exercise enhances mitochondrial resilience. ATF5 has been identified as a key regulator of the mitochondrial quality control process that is activated by exercise.^69^ It integrates stress signals to enhance proteostasis and organelle renewal.

Together, the integration of mitophagy and UPRmt signaling establishes a coordinated adaptive network that fortifies skeletal muscle against metabolic and proteotoxic stress, thereby sustaining mitochondrial bioenergetic efficiency, structural integrity, and cellular longevity.^64^^,^^69^

Caloric restriction enhances mitochondrial maintenance by promoting mitophagy through SIRT1-mediated deacetylation of PGC-1α and FOXO3. NAD+ precursors, such as NMN, boost NAD+ levels by 60%–80%. This reactivates sirtuins and poly(ADP-ribose) polymerases (PARPs), which repair oxidative DNA damage and restore mitochondrial function.^70^^,^^71^^,^^72^

Defects in mitochondrial biogenesis contribute to insulin resistance, metabolic inflexibility, and aging-related muscle loss (sarcopenia). This underscores the importance of interventions that target mitochondrial health, such as exercise, dietary changes, and medications.

#### Oxidative phosphorylation and ATP production

Mitochondria are the primary site of aerobic ATP production in skeletal muscle. They generate energy through oxidative phosphorylation using metabolic substrates from glycolysis, fatty acid β-oxidation, amino acid catabolism, and ketone body oxidation.

During glycolysis, pyruvate enters the mitochondrial matrix through the mitochondrial pyruvate carrier (MPC) and is then converted to acetyl-CoA by the pyruvate dehydrogenase (PDH) complex. Acetyl-CoA then enters the TCA cycle, generating NADH and FADH_2_, which donate electrons to the ETC. This process yields 30–32 ATP per glucose molecule, making it a highly efficient energy-generating pathway.^73^

Fatty acids undergo β-oxidation in the mitochondrial matrix. This process produces multiple acetyl-CoA units, as well as NADH and FADH_2_. These products then fuel the TCA cycle and ETC, respectively. During prolonged fasting or endurance exercise, fatty acid oxidation contributes more than 70% of total ATP production, making it the predominant energy source under low-glucose conditions.^74^

Glucogenic amino acids, such as alanine and glutamine, undergo deamination to produce intermediates like pyruvate or α-ketoglutarate. These intermediates then enter gluconeogenesis or replenish the TCA cycle through a process called anaplerosis. This process is essential for maintaining glucose homeostasis during fasting and supporting energy metabolism under catabolic stress.^75^

Ketone bodies, particularly β-HB and acetoacetate, are oxidized in skeletal muscle and other extrahepatic tissues. Acetoacetate is converted to acetyl-CoA, which enters the TCA cycle and supplies up to 70% of the brain’s energy demand during prolonged fasting. This metabolic shift spares glucose and reduces amino acid catabolism,^76^ thereby conserving lean muscle mass.

Dysregulation of mitochondrial oxidative phosphorylation impairs ATP production and increases ROS levels, resulting in oxidative stress, muscle dysfunction, and insulin resistance. Activation of AMPK and PGC-1α plays a pivotal role in restoring mitochondrial efficiency, promoting biogenesis, and reducing oxidative damage. This enhances metabolic resilience (Table 2).^77^

### Hormonal and cytokine regulation of skeletal muscle metabolism

**Figure 2:**
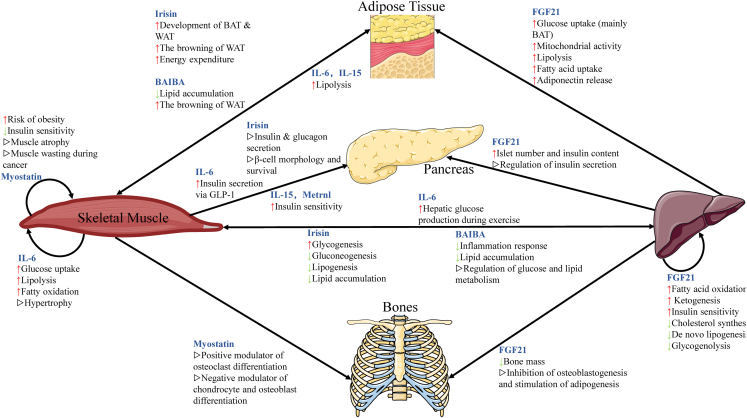
Myokine and cytokine signaling in skeletal muscle metabolism This schematic illustrates the concept of skeletal muscle as an endocrine organ, highlighting the release of selected exercise-induced myokines (e.g., IL-6, irisin, and FGF21) and their primary effects on peripheral tissues, as well as the impact of key pro-inflammatory cytokines on muscle. It focuses on this major signaling direction. The bidirectional nature of inter-tissue communication and a more comprehensive network of regulatory factors are discussed in detail in the text (see Sections hormonal and cytokine regulation of skeletal muscle metabolism and chronic inflammation and lipid metabolism dysregulation).

Skeletal muscle metabolism is intricately regulated by the dynamic interplay of hormones, myokines, and cytokines. These factors orchestrate processes such as glucose uptake, lipid oxidation, protein turnover, and mitochondrial function (Figure 2). These endocrine and paracrine signals ensure metabolic flexibility and systemic energy homeostasis in response to nutritional, physiological, and physical activity stimuli.

Insulin, a key anabolic hormone, promotes glucose uptake in skeletal muscle by activating the PI3K/Akt pathway, which facilitates GLUT4 translocation to the plasma membrane. Insulin also enhances glycogen synthesis by activating glycogen synthase and stimulates protein synthesis by activating mTORC1-mediated translational control. This supports nutrient storage and growth during the postprandial state.^21^^,^^77^^,^^78^

In contrast, glucagon mobilizes energy stores during fasting by stimulating hepatic glycogenolysis and gluconeogenesis via cAMP-PKA signaling, elevating circulating glucose levels. Catecholamines, such as epinephrine, activate β-adrenergic receptors in adipose tissue to promote lipolysis, releasing free fatty acids that serve as substrates for skeletal muscle β-oxidation during energy demand.^78^^,^^79^^,^^80^

Cortisol, a glucocorticoid hormone, supports fasting adaptation by upregulating PEPCK and G6Pase in the liver to drive gluconeogenesis. However, it also promotes skeletal muscle proteolysis through activation of the ubiquitin-proteasome system, providing amino acids for hepatic glucose production during catabolic stress.^79^^,^^81^

Emerging evidence positions bile acids as novel endocrine modulators of skeletal muscle. Beyond their classical digestive roles, bile acids activate the nuclear receptor FXR and membrane receptor TGR5 in muscle cells, triggering signaling cascades that enhance mitochondrial function, promote protein synthesis, and inhibit atrophy-related pathways. This gut-muscle axis represents a promising therapeutic target for counteracting muscle wasting in metabolic and aging-related conditions.^82^

Skeletal muscle acts as an endocrine organ that releases exercise-induced myokines. This endocrine communication is further extended by the release of small extracellular vesicles (sEVs). These vesicles carry proteins, lipids, and nucleic acids, allowing muscle to influence glucose and lipid metabolism in distant tissues like liver and adipose tissue, adding a novel layer to inter-organ crosstalk.^83^ For instance, IL-6 and irisin promote fatty acid oxidation and glucose uptake by activating the AMPK-PGC-1α signaling pathway. Meanwhile, FGF21 and BAIBA enhance mitochondrial function and anti-inflammatory responses through PPARα-dependent mechanisms (Figure 2).^84^ These myokines reinforce metabolic flexibility, support systemic insulin resistance, and protect against inflammation.

Conversely, pro-inflammatory cytokines, such as TNF-α and IL-1β, disrupt insulin signaling by promoting the serine phosphorylation of IRS-1. This impairs GLUT4 translocation. These cytokines also activate NF-κB, leading to muscle atrophy via the upregulation of proteolytic and ubiquitin-proteasome pathways.^85^ Additionally, myostatin, a TGF-β family member, negatively regulates muscle mass by inhibiting growth and promoting catabolism.

## Pathophysiology of metabolic dysregulation in skeletal muscle

Metabolic dysregulation in skeletal muscle is a multifactorial process. It is driven by genetic predispositions, environmental exposures, and sedentary lifestyle factors. These factors impair glucose utilization, lipid oxidation, and protein homeostasis. As the largest insulin-responsive organ, skeletal muscle plays a key role in systemic energy regulation and postprandial glucose disposal. Therefore, disruptions in its metabolic function significantly impair whole-body homeostasis, predisposing individuals to insulin resistance, T2D, sarcopenia, and related metabolic disorders.

**Figure 3:**
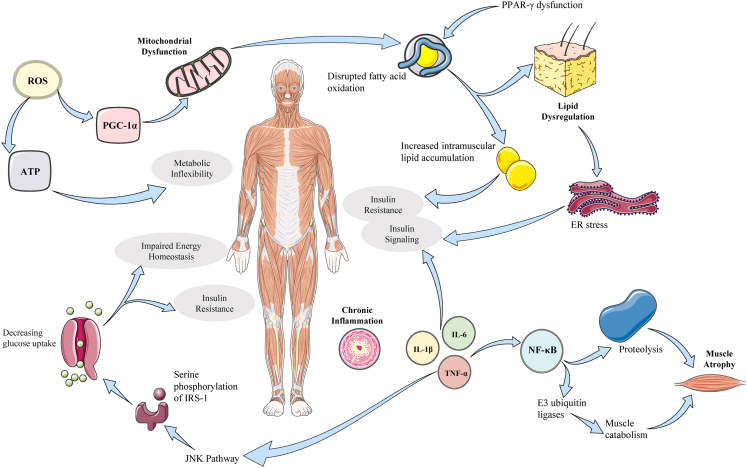
Pathological cycle linking mitochondrial dysfunction, inflammation, and lipid dysregulation to muscle wasting and metabolic disease This conceptual figure illustrates the proposed vicious cycle that perpetuates metabolic dysregulation in skeletal muscle. It highlights how core pathological domains—mitochondrial dysfunction (characterized by excessive ROS and bioenergetic deficit), chronic inflammation, and lipid dysregulation—interact to drive insulin resistance, metabolic inflexibility, and ultimately muscle wasting in conditions like type 2 diabetes and cachexia. The arrows represent dominant pathological influences within this cycle. It is important to note that molecules like ROS can have context-dependent roles; here, the focus is on their sustained, pathological excess. Detailed molecular pathways within each domain and their therapeutic targeting are discussed in Sections mitochondrial dysfunction and insulin resistance, chronic inflammation and lipid metabolism dysregulation, vicious cycle of metabolic dysregulation, muscle wasting disorders and metabolic dysregulation and therapeutic interventions.

Figure 3 illustrates how mitochondrial dysfunction, chronic inflammation, and lipid dysregulation synergistically promote muscle wasting and systemic insulin resistance. Mitochondrial impairments, marked by reduced biogenesis, elevated ROS, and compromised oxidative phosphorylation, diminish ATP production and metabolic flexibility. Meanwhile, inflammatory cytokines activate catabolic pathways, which accelerates proteolysis and insulin resistance. Additionally, lipid accumulation and impaired fatty acid oxidation induce lipotoxic stress, which further disrupts insulin signaling. These interconnected processes form a self-perpetuating cycle of energy imbalance, muscle atrophy, and metabolic decline. Understanding this cycle mechanistically is essential for developing interventions that preserve muscle function and prevent diseases like T2D and cachexia.

### Mitochondrial dysfunction and insulin resistance

As illustrated in Figure 3, mitochondrial dysfunction is a central pathological mechanism underlying skeletal muscle insulin resistance. This condition is characterized by reduced mitochondrial content, impaired oxidative phosphorylation, and defective mitochondrial biogenesis. These factors compromise cellular energy homeostasis.

A key feature of mitochondrial impairment is the overproduction of ROS, which damage mitochondrial DNA, lipids, and respiratory chain proteins. This oxidative stress disrupts ATP synthesis, induces mitochondrial fragmentation, and contributes to metabolic inflexibility. This limits the muscle’s ability to efficiently switch between fuel sources in response to energy demands.

PGC-1α, a transcriptional coactivator and master regulator of mitochondrial biogenesis and oxidative metabolism, is significantly downregulated in insulin-resistant states. This downregulation impairs mitochondrial renewal and adaptability, which further compounds energy deficits and reduces oxidative efficiency.

Concurrently, mitochondrial dysfunction impairs fatty acid oxidation, primarily by dysregulating CPT1, the rate-limiting enzyme for transporting fatty acids into mitochondria. This leads to the accumulation of intramyocellular lipids, contributing to lipotoxicity, ER stress, and the inhibition of insulin signaling via the activation of stress kinases and inflammatory mediators.

Furthermore, defective mitophagy, the process by which damaged mitochondria are degraded, prevents the removal of dysfunctional organelles. This perpetuates ROS accumulation and exacerbates metabolic dysregulation.

Parallel to these bioenergetic failures, intrinsic defects in muscle glucose sensing also contribute to insulin resistance. The recently identified factor PAGR1 acts as a transcriptional brake in high glucose, suppressing GLUT4 expression and translocation. Muscle-specific PAGR1 ablation enhances glucose uptake and protects against insulin resistance and hepatic steatosis, positioning it as a novel upstream pathogenic component.^86^ Importantly, insulin resistance in obesity may also originate outside muscle. Adipose tissue-derived adrenomedullin can induce insulin resistance in the vascular endothelium, impairing substrate delivery to muscle and thereby driving systemic metabolic dysfunction.^87^

These defects create a vicious cycle of mitochondrial ROS overproduction, lipid overload, insulin resistance, and impaired energy balance. This cycle contributes to the development of systemic metabolic disorders, including T2D. When evaluating mitochondrial dysfunction, it is essential to contrast animal and human findings. In rodent models of insulin resistance, AMPK and PGC-1α deficiencies are often reversible with interventions like exercise or drugs, whereas human clinical trials show mixed results, particularly in aged or comorbid populations. For example, AMPK activators like metformin exhibit efficacy in animals but variable outcomes in humans, partly due to pharmacokinetic and physiological differences.^23^^,^^88^ This cycle also contributes to muscle-wasting conditions such as sarcopenia and cachexia, which further aggravate metabolic instability (Figure 3).

### Chronic inflammation and lipid metabolism dysregulation

Chronic low-grade inflammation is a defining feature of metabolic dysfunction in skeletal muscle. It plays a pivotal role in developing insulin resistance, muscle catabolism, and energy imbalance. As shown in Figure 3, pro-inflammatory cytokines, such as TNF-α, IL-6, and IL-1β, activate stress-responsive signaling cascades, including the NF-κB and JNK pathways. These pathways impair insulin signaling and promote proteolytic muscle degradation.^89^^,^^90^

NF-κB activation upregulates muscle-specific E3 ubiquitin ligases, such as atrogin-1 and MuRF1. This drives ubiquitin-proteasome-mediated proteolysis and accelerates muscle atrophy. Concurrently, JNK signaling induces serine phosphorylation of IRS-1, disrupting insulin receptor signaling and exacerbating metabolic inflexibility.

This cytokine-driven inflammation is a central pillar in the development of sarcopenia. Sustained NF-κB and JNK activation not only impair insulin signaling but also directly upregulate muscle-specific E3 ubiquitin ligases like MuRF1 and Atrogin-1, accelerating proteasomal degradation of myofibrillar proteins and driving progressive muscle loss.^91^

In parallel with inflammatory stress, the dysregulation of lipid metabolism significantly contributes to insulin resistance in skeletal muscle. Impaired peroxisome proliferator-activated receptor gamma (PPARγ) activity impairs both fatty acid oxidation and lipid buffering capacity. This leads to the intracellular accumulation of lipotoxic intermediates, such as ceramides and DAGs. These molecules directly inhibit insulin signaling components and elicit ER stress, which further disrupts cellular homeostasis.^5^^,^^92^

Excess lipid deposition in muscle fibers impairs insulin action and provokes oxidative stress, which reinforces mitochondrial dysfunction and sustains a vicious cycle of inflammation, lipotoxicity, and insulin resistance (Figure 3). The bidirectional crosstalk between inflammatory signaling and lipid derangement exacerbates metabolic deterioration and predisposes individuals to muscle wasting syndromes, including sarcopenia, cachexia, and metabolic syndrome.

Strategies that target inflammation, restore lipid homeostasis, and enhance mitochondrial resilience show significant therapeutic potential in reversing insulin resistance and preserving skeletal muscle integrity under metabolic stress.

### Vicious cycle of metabolic dysregulation

Metabolic dysregulation in skeletal muscle perpetuates a vicious cycle involving mitochondrial dysfunction, chronic inflammation, and lipid dysregulation. This cycle accelerates metabolic decline and muscle degeneration (Figure 3).

Excessive production of ROS, coupled with impaired PGC-1α signaling, undermines mitochondrial biogenesis and oxidative efficiency. This shift in mitochondrial function increases reliance on anerobic glycolysis, resulting in elevated lactate levels and metabolic acidosis. These changes disrupt cellular energetics and impair muscle performance.

Concurrently, the persistent activation of inflammatory pathways, particularly NF-κB and JNK, sustains a pro-inflammatory microenvironment. This microenvironment exacerbates insulin resistance and stimulates proteolytic degradation via the ubiquitin-proteasome system. This process accelerates muscle atrophy.

Metabolically, PPAR-γ dysfunction impairs fatty acid handling and disrupts CPT1-mediated mitochondrial fatty acid oxidation. This results in lipid overload, ceramide accumulation, and lipotoxicity. These lipid intermediates interfere directly with insulin signaling and intensify mitochondrial stress, fostering metabolic inflexibility and systemic insulin resistance.

These interlinked processes reinforce one another, creating a downward spiral of energy imbalance, muscle wasting, and systemic metabolic impairment. The cumulative effects of oxidative stress, inflammatory signaling, and lipid toxicity form a pathological cascade that is central to the development of T2D, cachexia, and sarcopenia.

To interrupt this cycle, integrative therapeutic strategies must target mitochondrial health, inflammatory modulation, and lipid metabolism to restore skeletal muscle function and metabolic resilience (Figure 3).^93^

### Muscle wasting disorders and metabolic dysregulation

Muscle wasting disorders, such as cachexia, sarcopenia, disuse atrophy, and muscular dystrophies, are characterized by the progressive loss of skeletal muscle mass and function. These disorders are accompanied by chronic inflammation, excessive proteolysis, and mitochondrial dysfunction. These conditions result in metabolic decline, functional impairment, and an increased risk of systemic diseases (Figure 3).

They commonly arise from disrupted protein turnover, altered substrate metabolism, and a sustained proinflammatory environment. These factors together exacerbate insulin resistance and metabolic inflexibility, forming a pathological feedback loop. The major molecular contributors have been outlined.

Mitochondrial dysfunction: deficient mitochondrial biogenesis, increased production of ROS, and impaired mitophagy result in cumulative mitochondrial damage and reduced oxidative capacity. Notably, dysregulation of mitochondrial calcium (Ca^2+^) handling also contributes to age-related muscle decline. Diminished Ca^2+^ uptake through the mitochondrial calcium uniporter (MCU) impairs the rapid matching of ATP production to demand in aged muscle. Intriguingly, the natural compound oleuropein can allosterically activate MCU, restoring mitochondrial Ca^2+^ uptake, oxidative function, and physical performance in aged mice,^94^ highlighting a novel target for intervention. The downregulation of PGC-1α, a master regulator of mitochondrial renewal and energy metabolism, further disrupts energy homeostasis and worsens skeletal muscle impairment (Figure 3).

Chronic inflammation and proteolysis: sustained elevations in proinflammatory cytokines, such as TNF-α, IL-6, and IL-1β, activate catabolic pathways including NF-κB and JNK, which stimulates E3 ubiquitin ligases such as atrogin-1 and MuRF1. Together, these pathways accelerate the proteasome-mediated degradation of structural muscle proteins, contributing to atrophy, fibrosis, and functional decline (Figure 3).

Lipid dysregulation: disrupted PPAR-γ signaling impairs fatty acid oxidation and promotes lipid accumulation within skeletal muscle fibers. This lipid overload not only triggers lipotoxicity and ER stress but also amplifies insulin resistance, diminishing metabolic flexibility and perpetuating systemic metabolic dysfunction (Figure 3). Furthermore, integrated multi-omics analyses reveal that sarcopenia is characterized by a specific blockade in BCAA catabolism, leading to the accumulation of detrimental intermediates. Restoring this catabolic flux in models ameliorates muscle atrophy, positioning BCAA metabolism as a causal node and therapeutic target in sarcopenia.^95^

In summary, these interdependent processes create a cycle of self-sustaining muscle degradation and metabolic deterioration. Understanding the underlying mechanisms of muscle wasting disorders is essential for developing targeted interventions that preserve muscle mass, restore metabolic resilience, and improve outcomes in chronic metabolic and neuromuscular diseases.

## Therapeutic interventions

**Figure 4:**
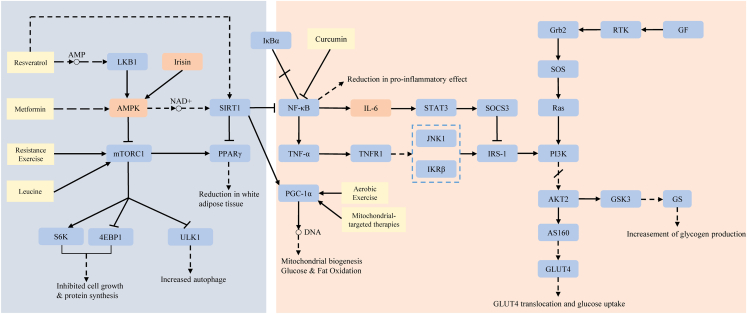
Therapeutic targets in skeletal muscle metabolism This figure highlights key therapeutic targets in skeletal muscle, including AMPK, myokines, and mitochondrial pathways, along with interventions such as exercise, pharmacological agents, and nutritional supplements. It illustrates the interplay between these targets and metabolic pathways, emphasizing their roles in enhancing muscle metabolism, improving insulin sensitivity, reducing inflammation, and promoting muscle growth.

Recent advances in skeletal muscle biology have enabled the development of targeted interventions that restore metabolic balance and combat chronic diseases, such as obesity, T2D, and metabolic syndrome. Because skeletal muscle plays a pivotal role in regulating glucose and lipid homeostasis, therapeutic strategies focus on enhancing mitochondrial function, stimulating energy-sensing pathways, reducing inflammation, and promoting muscle anabolism. Figure 4 illustrates key therapeutic targets, including AMPK activation, myokine signaling, and mitochondrial regulators. These pathways can be modulated through exercise, pharmacological agents, and nutritional interventions to improve insulin resistance and muscle metabolism. Table 3 provides an overview of these strategies, highlighting their mechanisms of action, clinical benefits, and limitations.

**Table 3:** Overview of therapeutic intervention categories targeting skeletal muscle metabolism

Therapeutic strategies		Mechanism of action	Clinical evidence	Potential benefits	Limitations for each intervention	Reference
Pharmacological agents	Metformin	Activates AMPK, which increases glucose uptake and fat burning in muscle.	Widely used for managing metabolic diseases. Emerging evidence for benefits in non-diabetic populations with metabolic syndrome.	Enhances insulin resistance and metabolic flexibility. Reduces risk of cardiovascular complications in diabetic patients.	Side effects include nausea, diarrhea, and abdominal discomfort; Contraindicated in renal impairment. Limited effects on weight loss and muscle mass preservation.	DeFronzo et al.^15^; Viollet et al.^88^
	Thiazolidinediones (TZDs)	PPAR-γ, promoting fatty acid storage in adipose tissue and enhancing insulin resistance in muscle.	Demonstrated to improve glycemic control and reduce insulin resistance in T2DM patients.	Enhances insulin resistance, mitigates lipotoxicity.	Cardiovascular risks, bladder cancer concerns, bone density loss with long-term use.	Yki-Jarvinen et al.^96^; Wu et al.^12^
	Compounds enhancing mitochondrial function (e.g., resveratrol, coenzyme Q10)	Activates PGC-1α, enhancing mitochondrial growth and function, helping muscles adapt to energy demands more effectively.	Shows promise in stabilizing mitochondrial membranes and boosting energy production.	Improves muscle’s ability to meet energy demands and reduce oxidative stress.	Clinical efficacy varies; optimal dosing and long-term effects unclear.	Allard et al.^97^; Zhou et al.^10^
	Mitochondrial-targeted drugs (e.g., elamipretide)	Stabilizes mitochondrial membranes, boost energy production, and reduce oxidative stress in metabolic diseases.	Newer drugs with promising results in early studies.	May improve metabolic health by enhancing mitochondrial function.	As they are newer, long-term safety and efficacy data might be limited.	Parikh et al.^98^
	Anti-inflammatory therapies (e.g., cytokine inhibitors, NSAIDs, curcumin)	Targets inflammatory pathways to reduce muscle inflammation and improve insulin resistance.	Curcumin has shown potential in clinical studies to lower inflammation and improve metabolic profiles.	May reduce inflammation and enhance insulin resistance.	The document does not specify limitations, but anti-inflammatory drugs can have side effects and may not be suitable for all patients.	Andelid et al.^99^; Wu et al.^12^
	Anabolic agents for muscle growth (e.g., SARMs, growth hormone secretagogue)	Designed to enhance muscle growth and prevent muscle wasting.	Myostatin inhibitors like bimagrumab have shown potential benefits in treating sarcopenia and muscle wasting disorders.	Increases muscle mass and strength. Improves the muscle’s ability to absorb glucose and fats.	Limited improvements in muscle strength or exercise capacity; potential immune responses.	Solomon et al.^100^
	Myostatin inhibitors (e.g., bimagrumab)	Inhibition of myostatin to counteract muscle wasting and improve metabolic health.	Phase II trial showed increased muscle mass and improved performance in sarcopenia.	Effective against muscle wasting and improves metabolic health.	Side effects include immune responses; limited functional improvements.	Rooks et al.^101^
Exercise	Aerobic exercise	Activates AMPK/PGC-1α pathways, enhancing mitochondrial function and fatty acid oxidation.	Strong evidence for improved insulin resistance and metabolic health.	Enhances glucose uptake and fatty acid oxidation. Promotes weight loss and cardiovascular benefits.	Requires long-term adherence and lifestyle changes. Variable individual responses depending on genetics, age.	Wu et al.^12^; Richter et al.^1^
	Resistance training	Stimulates mTORC1 pathway, increasing muscle protein synthesis. Enhances glucose uptake and increases muscle mass, contributing to improved metabolic rate.	Boosts muscle’s capacity for glucose and fat metabolism. Improves glycemic control, muscle strength, and metabolic health, particularly in older adults and those with insulin resistance.	Increases muscle mass and strength. Reduces age-related muscle loss (sarcopenia). Increases IL-15 secretion, which promotes muscle hypertrophy and enhances glucose uptake through AMPK signaling	Risk of injury, particularly in older adults or those with pre-existing conditions.Requires access to proper equipment and guidance for safe performance.	Philp et al.^102^; Zunner et al.^103^
Nutritional interventions	High-protein diet	Provides essential amino acids, particularly leucine, which activates the mTORC1 pathway to stimulate muscle protein synthesis.	BCAA supplementation enhances muscle recovery and reduces soreness in resistance training.	Promotes muscle growth and repair. Improves satiety and aids in weight management. Supports metabolic health by enhancing muscle metabolism.	Potential renal strain in individuals with pre-existing kidney disease. Requires careful macronutrient balance to avoid excessive calorie intake.	Phillips et al.^104^; Jackman et al.^105^; Verreijen et al.^106^
	Omega-3 fatty acids	Enhances metabolic flexibility by allowing muscles to switch between carbohydrates and fats for energy. Reduces inflammation by lowering levels of pro-inflammatory cytokines and oxidative stress.	The role of omega-3 fatty acids in managing insulin resistance and metabolic syndrome. EPA and DHA significantly reduced plasma triglycerides in obese individuals with insulin resistance.	Reduces inflammation and oxidative stress. Improves insulin resistance. Lowers triglyceride levels. Supports metabolic health and management of muscle-related metabolic disorders.	Benefits may vary depending on dosage, duration, and individual metabolic status.	Calder et al.^107^; You et al.^108^; Wu et al.^12^

This table summarizes the major categories of therapeutic interventions, their general mechanisms of action, and associated clinical benefits and limitations. The “clinical evidence” column qualitatively reflects the overall strength of supporting research (e.g., “strong evidence,” “emerging evidence,” or “preclinical data”). Detailed information on study designs, sample sizes, statistical outcomes, and trial phases can be found in the cited primary literature and in the corresponding text sections (Sections pharmacological interventions in skeletal muscle, exercise as therapy, nutritional interventions, and clinical perspectives and translational medicine).”

### Pharmacological interventions in skeletal muscle

Pharmacological strategies that target skeletal muscle metabolism aim to restore insulin resistance, enhance mitochondrial function, suppress chronic inflammation, and promote muscle growth. Metformin and thiazolidinediones (TZDs) improve insulin resistance primarily through AMPK activation and modulation of lipid metabolism. However, their clinical use may be limited by gastrointestinal side effects, and TZDs carry additional risks, such as cardiovascular issues and fluid retention.^96^ Figure 4 depicts therapeutic targets such as AMPK, myokine signaling, and mitochondrial modulators, which are key nodes in metabolic regulation and are summarized in Table 3.

Addressing mitochondrial dysfunction is central to pharmacological interventions. Notably, GLP-1 receptor agonists (GLP-1 RAs), beyond their glycemic and weight effects, may directly benefit skeletal muscle. Evidence suggests they can enhance mitochondrial biogenesis and oxidative capacity while reducing inflammation, indicating potential repurposing for muscle-wasting conditions.^109^ Therapeutics such as PGC-1α activators, mitophagy inducers, and biogenesis enhancers aim to restore ATP production, improve oxidative phosphorylation, and reestablish metabolic flexibility.^110^ Compounds such as resveratrol, coenzyme Q10, and elamipretide have been shown to increase mitochondrial efficiency and skeletal muscle energetics.^111^^,^^112^

Anti-inflammatory pharmacotherapies also offer substantial benefits. Suppressing key inflammatory pathways, such as NF-κB and JNK, mitigates cytokine-induced proteolysis and insulin resistance. Although agents such as corticosteroids and TNF-α inhibitors have shown promise, their long-term use is limited due to systemic side effects. Curcumin, a nutraceutical, has emerged as a promising candidate capable of downregulating inflammatory cytokines and improving insulin signaling with fewer adverse effects.

Anti-inflammatory strategies play a key role in reducing muscle degradation and systemic metabolic dysfunction. Pharmacological agents, including corticosteroids, cytokine inhibitors, and modulators of the NF-κB and JNK pathways, can suppress chronic inflammation and delay disease progression. However, concerns remain regarding their long-term safety and systemic effects.^113^ Curcumin, an emerging alternative, demonstrates promise as a bioactive compound that reduces pro-inflammatory signaling and enhances insulin resistance. It offers a safer adjunctive approach for managing muscle-related metabolic impairments.^114^

The pharmacological modulation of skeletal muscle metabolism is further advanced by agents such as PPARγ agonists, carnitine palmitoyltransferase 1 (CPT1) activators, and AMPK enhancers. These agents collectively enhance substrate utilization, lipid oxidation, and glycemic control. These compounds support metabolic flexibility and mitochondrial efficiency. Pharmacological targeting of AMPK and mTOR pathways illustrates translational gaps. Animal studies frequently report pronounced benefits from AMPK activators (e.g., AICAR) or mTOR inhibitors, but human trials encounter challenges such as off-target effects and variable efficacy. For instance, mTOR inhibitors like rapamycin show robust effects in rodent muscle atrophy models, yet human applications are limited by immunosuppression and metabolic side effects, highlighting the need for species-tailored therapeutic strategies.^25^^,^^113^ Anabolic agents, notably selective androgen receptor modulators (SARMs) and myostatin inhibitors, hold therapeutic promise for preserving muscle mass and promoting hypertrophy in muscle-wasting disorders. However, challenges such as immune activation, off-target effects, and limited improvements in functional outcomes highlight the necessity of continued optimization and clinical validation.^101^^,^^115^

Together, these pharmacological interventions, which target mitochondrial bioenergetics, inflammation, and anabolic signaling, provide a comprehensive framework for reversing skeletal muscle metabolic dysfunction and improving systemic metabolic health (Figure 4; Table 3).

### Exercise as therapy

Exercise is a fundamental non-pharmacological intervention that enhances skeletal muscle metabolism, improves insulin resistance, promotes lipid oxidation, and maintains systemic energy balance. As shown in Figure 4, exercise activates several key regulatory pathways, including AMPK signaling, mitochondrial biogenesis, and myokine secretion. These pathways play a crucial role in preventing metabolic dysfunction and promoting muscle health.

Aerobic exercise, such as walking or running, stimulates mitochondrial oxidative capacity via PGC-1α activation. This enhances glucose and fatty acid oxidation, improving mitochondrial efficiency.^116^ Resistance training, including weight lifting, promotes mTORC1 signaling, which leads to muscle hypertrophy and increased substrate utilization. Resistance training also increases basal metabolic rate, which further supports long-term energy expenditure and metabolic health.^102^

Importantly, resistance exercise significantly upregulates the secretion of IL-15, a myokine associated with muscle protein synthesis and glucose uptake through AMPK-mediated pathways.^103^ IL-15 also facilitates skeletal muscle-adipose tissue crosstalk, promoting muscle growth and enhancing metabolic flexibility and insulin resistance, particularly relevant for individuals with obesity or T2D.

As summarized in Table 3, the combination of aerobic and resistance exercise optimizes muscle substrate metabolism, enhances hormonal signaling, and reduces chronic inflammation. This makes it a powerful and accessible strategy for preventing and managing metabolic disorders, including T2D, sarcopenia, and metabolic syndrome.

Regular exercise has robust therapeutic effects on metabolic disorders. It enhances insulin resistance, glucose uptake, and lipid metabolism while promoting metabolic flexibility. Mechanistically, physical activity increases GLUT4 expression in skeletal muscle, thereby improving postprandial glucose clearance. Additionally, physical activity suppresses inflammation by decreasing pro-inflammatory cytokines, such as TNF-α, and increasing anti-inflammatory mediators, such as IL-10. These adaptations mitigate insulin resistance and support long-term metabolic homeostasis. Meta-analyses show that a combination of aerobic and resistance exercise significantly improves glycemic control and reduces insulin resistance more effectively than sedentary interventions do in individuals with T2D and metabolic syndrome^117^^,^^118^ (Figure 4; Table 3).

Despite the well-established benefits of exercise, long-term adherence to exercise regimens remains a challenge, especially for individuals with chronic diseases. Barriers to adherence include time constraints, low motivation, comorbid conditions, and physical limitations. Furthermore, excessive or improperly structured exercise can lead to overuse injuries, joint degeneration, and musculoskeletal complications, particularly in older adults or individuals with preexisting frailty. Therefore, personalized exercise prescriptions guided by trained health professionals are essential to optimize outcomes, minimize adverse effects, and ensure sustainable engagement in physical activity across diverse patient populations (Table 3).

### Nutritional interventions

Nutritional strategies play a critical role in optimizing skeletal muscle metabolism, supporting muscle growth, and improving metabolic health. A balanced intake of high-quality protein, BCAAs, and omega-3 fatty acids is particularly beneficial for individuals with metabolic disorders, muscle wasting, or sarcopenia. Integrating personalized nutrition with exercise and pharmacological treatments offers a comprehensive strategy for improving metabolic health (Figure 4).

Nutritional interventions are essential for enhancing skeletal muscle metabolism, promoting anabolic signaling, and supporting systemic metabolic health. The strategic intake of high-quality protein, BCAAs, and omega-3 polyunsaturated fatty acids (PUFAs) has been shown to stimulate muscle protein synthesis, reduce inflammation, and improve insulin resistance, particularly in populations affected by sarcopenia, cachexia, or T2D. As shown in Figure 4, these nutrients modulate key metabolic pathways, including mTORC1 activation, PGC-1α expression, and anti-inflammatory signaling. This reinforces muscle integrity and energy balance. Combined with exercise and pharmacological therapies, personalized nutrition is a cornerstone of multimodal strategies that aim to restore metabolic flexibility and preserve muscle mass in aging and diseased states (Table 3).

#### Protein and amino acids

Amino acids are essential modulators of skeletal muscle metabolism. They influence protein synthesis, immune regulation, and energy homeostasis. Targeted amino acid supplementation supports muscle maintenance and metabolic resilience, particularly during periods of catabolic stress. Glutamine, for example, enhances immune function and promotes muscle recovery following injury or illness,^119^ while arginine improves endothelial function through NO production,^120^ thereby enhancing blood flow and nutrient delivery. Citrulline, an arginine precursor, has been shown to promote muscle protein synthesis, especially in malnourished or sarcopenic populations.^121^

Leucine-enriched BCAAs play a pivotal anabolic role by activating mTORC1 signaling pathway, thereby stimulating muscle protein synthesis and improving metabolic efficiency. In the context of resistance exercise and recovery, consuming protein after exercise increases muscle repair and improves functional adaptation.^102^ Studies have shown that BCAA supplementation reduces exercise-induced muscle soreness, accelerates recovery, and improves training outcomes, particularly among athletes and individuals undergoing physical rehabilitation.^105^

However, a comprehensive understanding of BCAAs must also encompass their complex role as metabolic signaling molecules beyond direct anabolic stimulation. Recent syntheses emphasize that while leucine potently activates mTORC1 to drive hypertrophy, all BCAAs collectively influence systemic metabolism by modulating insulin sensitivity and mitochondrial function. It is crucial to note that chronic, excessive elevation of BCAA levels—a common feature in obesity—can paradoxically contribute to insulin resistance. This detrimental effect is mediated through sustained mTORC1 overactivation and the accumulation of incomplete metabolites, such as BCKAs, which interfere with insulin signaling. This duality underscores the critical importance of achieving a precise balance in BCAA availability to harness their therapeutic benefits while avoiding metabolic dysregulation.^122^

Further clinical studies underscore the benefits of leucine-rich supplementation in preserving muscle mass and enhancing glucose metabolism among older adults, who are particularly vulnerable to muscle wasting and insulin resistance.^106^ As illustrated in Figure 4, amino acid supplementation interacts with essential regulatory pathways, including mTORC1 activation and myokine signaling. This interaction provides a nutritionally accessible method for optimizing metabolism. Table 3 provides a detailed summary of these interventions, including their mechanisms, clinical applications, and limitations.

#### Omega-3 fatty acids

Omega-3 PUFAs, particularly EPA and docosahexaenoic acid (DHA), have many benefits for skeletal muscle metabolism and systemic metabolic health. These bioactive lipids enhance mitochondrial efficiency, reduce oxidative stress, and mitigate chronic, low-grade inflammation. This supports muscle preservation and insulin resistance.^107^

Omega-3s have several mechanistic effects. They lower circulating pro-inflammatory cytokines, such as TNF-α and IL-6; downregulate NF-κB signaling; and modulate lipid metabolism by improving fatty acid oxidation and reducing ectopic lipid deposition. These effects contribute to improved metabolic flexibility and glycemic control, particularly in individuals with insulin resistance, obesity, or T2D. Furthermore, EPA and DHA stimulate muscle protein synthesis via mTORC1 and Akt signaling, which supports anabolic responses in aging and muscle-wasting conditions.

As illustrated in Figure 4, omega-3s engage key therapeutic pathways including inflammation resolution, mitochondrial remodeling, and substrate utilization. Clinical applications and outcome data summarized in Table 3 highlight their role as adjunctive nutritional therapies in the management of metabolic disorders, sarcopenia, and cachexia.

#### Challenges and limitations

Excessive protein intake may increase the risk of kidney injury, especially in individuals with kidney disease, emphasizing the need for individualized nutritional plans.^123^ While BCAA supplementation supports muscle recovery, excessive intake may disrupt amino acid balance and contribute to insulin resistance, requiring careful dosing and monitoring.^124^

Although nutritional interventions show therapeutic promise, several challenges must be addressed to ensure safety and efficacy. For example, excessive protein intake may elevate the risk of kidney injury, especially in individuals with pre-existing renal impairment. This underscores the importance of individualized dietary planning and medical supervision.^123^ Similarly, while BCAA supplementation supports muscle recovery and anabolic signaling, overconsumption may disrupt amino acid homeostasis and has been linked to insulin resistance via the accumulation of BCKAs and altered mitochondrial metabolism.^124^

To maximize the metabolic and anabolic benefits of amino acid-based therapies while minimizing adverse effects, it is essential to achieve an optimal balance of protein quality, timing, and quantity. Figure 4 illustrates how integrating nutritional strategies with pharmacological and exercise-based approaches can target multiple regulatory nodes in skeletal muscle metabolism, including AMPK, mTORC1, and inflammatory signaling.

Looking ahead, future research should investigate synergistic interventions that combine nutrition with mitochondrial-targeted therapies, myokine modulation, and genetic tools such as gene editing to enhance metabolic plasticity and long-term muscle health. These multi-target strategies, summarized in Table 3, may offer innovative pathways to prevent or reverse metabolic disease progression.

## Clinical perspectives and translational medicine

While preclinical models have been instrumental in elucidating the fundamental mechanisms of skeletal muscle metabolism, translating these findings into effective human therapies presents significant challenges. This section critically examines the human clinical evidence for key regulatory pathways and discusses the translational limitations of preclinical data in the context of diabetes, obesity, and sarcopenia.

### Human evidence for key metabolic pathways

The core signaling networks involving AMPK, mTOR, and PGC-1α, while conserved, exhibit critical differences in their regulation and response in humans compared to animal models.

AMPK signaling: in human skeletal muscle, exercise is a potent physiological activator of AMPK. Acute aerobic exercise increases AMPK phosphorylation and activity, which correlates with increased glucose uptake and fatty acid oxidation.^125^ However, the responsiveness of AMPK can be blunted in insulin-resistant states. For instance, while metformin, a known AMPK activator, improves glycemic control in patients with T2D, its direct effects on skeletal muscle AMPK in humans are less pronounced than in rodent models, suggesting that its primary mechanism may involve indirect or tissue-specific effects.^126^ This highlights a key translational gap where the robustness of AMPK activation seen in preclinical studies does not fully translate to human pathophysiology.

mTORC1 signaling: resistance exercise robustly activates mTORC1 signaling and stimulates muscle protein synthesis in humans, a response that is essential for muscle hypertrophy.^78^ However, the anabolic response to amino acids, particularly leucine, is often attenuated in older adults, a condition known as anabolic resistance. This phenomenon is a major contributor to sarcopenia and is not as prominently observed in young rodent models, underscoring the importance of studying aged human populations.^127^ Furthermore, while BCAA supplementation can enhance mTORC1 signaling, chronic elevation in plasma BCAAs is paradoxically associated with an increased risk of insulin resistance and T2D in large human cohort studies, a complex relationship not fully captured in standard animal feeding experiments.^49^

PGC-1α and mitochondrial biogenesis: endurance exercise is a powerful inducer of PGC-1α expression and mitochondrial biogenesis in human skeletal muscle. Training increases mitochondrial density and oxidative capacity, which is a cornerstone for improving metabolic health in conditions like obesity and T2D. However, the magnitude of PGC-1α induction in humans is generally more modest and variable than the dramatic upregulation often reported in rodent studies. This variability is influenced by factors such as exercise intensity, duration, genetic background, and age.^128^ For example, the efficacy of pharmacological PGC-1α activators like resveratrol in improving mitochondrial function in humans has been inconsistent, with some studies showing minimal effects, thereby highlighting the challenges in replicating preclinical successes in clinical trials.^129^

### Translational limitations and the path forward

Discrepancies between animal and human studies stem from a variety of biological and methodological factors. Preclinical models typically use genetically uniform young animals that are kept in controlled environments. This approach fails to capture the genetic, environmental, and physiological diversity found in human populations. Furthermore, inducing disease in animals through methods such as feeding them a high-fat diet or pharmacologic manipulation only partially replicates the complex, multifactorial etiology of human metabolic syndromes, which evolve gradually over the lifespan.

To bridge this translational gap, future research must prioritize human-centric approaches. This includes the aforementioned key steps.

1. Larger, well-controlled human trials area required that incorporate biomarker validation (e.g., imaging for intramyocellular lipids and measures of mitochondrial function) to confirm target engagement.
2. Stratifying patients based on age, sex, genetic predisposition, and disease severity is essential to identify responders and non-responders to interventions.
3. Advanced omics technologies should be used on human muscle biopsies to define molecular signatures of diseases and treatment responses, moving beyond reliance on rodent transcriptomics alone.

In summary, preclinical research is invaluable for elucidating molecular mechanisms; however, effective translation to human health requires systematic validation in clinical contexts. It is essential to recognize and address these translational constraints to advance precision-based therapies that target the molecular underpinnings of diabetes, obesity, and sarcopenia. This will ultimately improve metabolic health across diverse human populations.

## Future research directions

Integrative multi-omics approaches are critical for mapping the molecular networks underlying exercise adaptation. A recent phosphoproteomic and acetylproteomic study in human skeletal muscle revealed distinct, modality-specific phosphorylation and acetylation signatures induced by aerobic versus resistance exercise, highlighting coordinated modifications in metabolic and anabolic pathways.^130^ This work exemplifies how profiling the dynamic “modifome” can uncover novel, exercise-regulated signaling nodes, advancing our understanding of muscle plasticity and identifying potential targets for metabolic therapeutics.

Future investigations into skeletal muscle metabolism should employ advanced omics technologies, including genomics, transcriptomics, proteomics, and metabolomics, to achieve a comprehensive and integrative understanding of skeletal muscle function, adaptation, and disease mechanisms. Applying single-cell omics approaches will further elucidate cell-type-specific metabolic adaptations and pathological alterations, facilitating the identification of novel biomarkers for muscle health and disease.^131^^,^^132^^,^^133^ There is emerging evidence on exosomal microRNAs (miRNAs) derived from skeletal muscle that underscores their pivotal role in muscle-adipose tissue crosstalk. This evidence suggests that they have the potential to be used as biomarkers of metabolic status and as therapeutic targets for promoting muscle regeneration.^134^

Recent work demonstrates that tissue-derived mitochondria-rich extracellular vesicles (Ti-mitoEVs) can deliver functional mitochondrial components to recipient cells, enhancing biogenesis and oxidative capacity.^135^ This approach represents a novel strategy to directly address mitochondrial dysfunction in skeletal muscle, with potential applications in treating sarcopenia, cachexia and metabolic myopathies through endogenous repair mechanisms.

Future studies should examine how muscle adapts to different types of exercise, focusing on important regulatory nodes such as PGC-1α, mTOR, and AMPK. These studies should also explore the coordinated roles of these nodes in autophagy and apoptosis to inform the development of personalized exercise interventions. Exploring myokines (e.g., irisin and myostatin) and their intricate communication with adipose tissue, the liver, and the immune system will provide valuable insights into novel molecular targets for therapeutic modulation.

Furthermore, advancing research on age-related muscle atrophy, mitochondrial dysfunction, and the impact of lifestyle and environmental factors is crucial for developing personalized preventive and restorative strategies. Promising avenues include interventions that modulate mitochondrial dynamics and biogenesis, such as nicotinamide riboside supplementation and exercise mimetics.^60^^,^^136^

Integrating personalized medicine with omics-driven approaches enables the design of precision-based interventions that are tailored to genetic, epigenetic, and environmental determinants. These interventions ultimately optimize skeletal muscle function and systemic metabolic health. Advances in these fields will accelerate the development of novel therapeutic strategies to improve muscle performance, resilience, and overall metabolic health.

## Conclusion

This review integrates the current understanding of complex regulatory networks that govern skeletal muscle metabolism. It emphasizes that metabolic flexibility, which is coordinated by AMPK, mTOR, and PGC-1α signaling, is a central determinant of systemic metabolic health. Moving beyond descriptive summaries, we critically analyze how disruptions to this regulatory triad lead to insulin resistance, lipid imbalance, and muscle wasting in metabolic disorders such as T2D, obesity, and sarcopenia. We also highlight the multifaceted role of skeletal muscle as an endocrine organ. Myokines, such as IL-6, irisin, and FGF21, mediate cross-organ communication with the liver, adipose tissue, and the immune system. This communication fundamentally shapes whole-body energy homeostasis and disease progression.

Despite these advances, substantial knowledge gaps persist, especially regarding the translation of mechanistic insights from preclinical models into effective human interventions. This review identified several translational barriers, including species-specific differences in signaling responses and context-dependent regulation of AMPK and PGC-1α. These barriers highlight the need for human-centered research approaches that account for genetic, environmental, and physiological variability. Progress in this field depends on refining model systems and answering critical questions that bridge basic mechanisms with clinical application. Key future directions include following agendas.

Defining the fiber-type-specific role of PGC-1α in sarcopenia requires further research. Although PGC-1α is recognized as a master regulator of oxidative metabolism, its protective function in fast-twitch (type II) fibers—which are preferentially lost during aging and sarcopenia—remains poorly defined. Future studies employing fiber-type-specific PGC-1α knockout models and single-nucleus RNA sequencing of human muscle biopsies could determine whether enhancing PGC-1α selectively in type II fibers preserves muscle mass and function.^137^

Establishing a metabolic signature for cachexia prediction and intervention is another crucial priority. The catabolic cycle characteristic of cachexia is perpetuated by the synergistic interplay between mitochondrial dysfunction, inflammation, and lipid dysregulation. A crucial research priority is identifying a circulating metabolite and myokine signature that predicts disease onset and therapeutic responsiveness. These biomarkers could guide the development of combined treatment strategies, such as pairing mitochondrial stabilizers with anti-cytokine agents, to disrupt the progression of muscle wasting.^13^

These emerging research avenues highlight a shift in the field of physiology, moving from a descriptive approach to a more precise, mechanism-based one. This shift paves the way for more effective strategies to prevent and treat skeletal muscle-related metabolic diseases.

How do nutrient-sensing pathways integrate to fine-tune metabolic flexibility in humans? The dynamic interplay among AMPK, mTOR, and sirtuin signaling in response to combined exercise and nutritional interventions, such as time-restricted feeding or intermittent fasting, remains incompletely understood in human skeletal muscle. Rigorously designed, well-controlled clinical trials are essential to delineate how these nutrient-sensing pathways cross-regulate energy metabolism, fuel selection, and insulin sensitivity. These studies will clarify how lifestyle interventions can harness these molecular networks to restore metabolic balance in real-world conditions.^27^

What is the therapeutic potential of targeting myokine networks? Beyond well-known mediators such as IL-6, irisin, and FGF21, the discovery of new exercise-induced myokines is a promising area of translational research. One major challenge and opportunity is determining whether it is possible to develop safe and effective therapeutics for metabolic syndrome, sarcopenia, and cachexia using recombinant analogs of beneficial myokines (e.g., BAIBA) or antagonists of deleterious ones (e.g., myostatin).^84^ Defining their regulatory mechanisms and inter-organ signaling networks is critical to transforming these molecular insights into clinical innovations.

In conclusion, the future of skeletal muscle research lies in integrating multi-omics data from well-characterized human cohorts with insights from advanced animal models. This approach bridges the gap between molecular mechanisms and clinical application. Future studies that address specific mechanistic questions and use systems-level approaches can advance beyond understanding disease pathology to developing precision therapies that restore metabolic resilience, enhance muscle function, and extend healthspan.

As the largest and most metabolically active organ, skeletal muscle is at the center of whole-body energy regulation. Through its control of glucose uptake, lipid oxidation, and amino acid metabolism, as well as its role as an endocrine organ that secretes myokines influencing the liver, adipose tissue, and immune system, skeletal muscle is not only a determinant of physical performance, but also a master regulator of systemic metabolic health.

## Acknowledgments

This study was supported by the 10.13039/501100001809National Natural Science Foundation of China (no. 32271496) and Research Foundation for Middle-aged and Young Scientists of Fujian Province (no. 2021ZQNZD005).

## Author contributions

D.L.: conceptualization, writing – original draft, writing – review & editing, and funding acquisition; L.Z.: writing – original draft and writing – review and editing; C.H.: contribution to discussion and writing – review and editing; W.S.: contribution to discussion and writing – review and editing.

## Declaration of interests

The authors have declared that no conflict of interest exists.
